# Supplementary material for: Reconciling persistent and dynamic hypotheses of working memory coding in prefrontal cortex
Source: Nat Commun. 2018 Aug 29;9:3498. doi: 10.1038/s41467-018-05873-3 (PMC6115433; doi:10.1038/s41467-018-05873-3)
Supplement: Supplementary file 1 — Supplementary Information [file 41467_2018_5873_MOESM1_ESM.pdf]

Supplementary Information

Reconciling persistent and dynamic hypotheses of working memory coding in prefrontal cortex

Cavanagh et al.

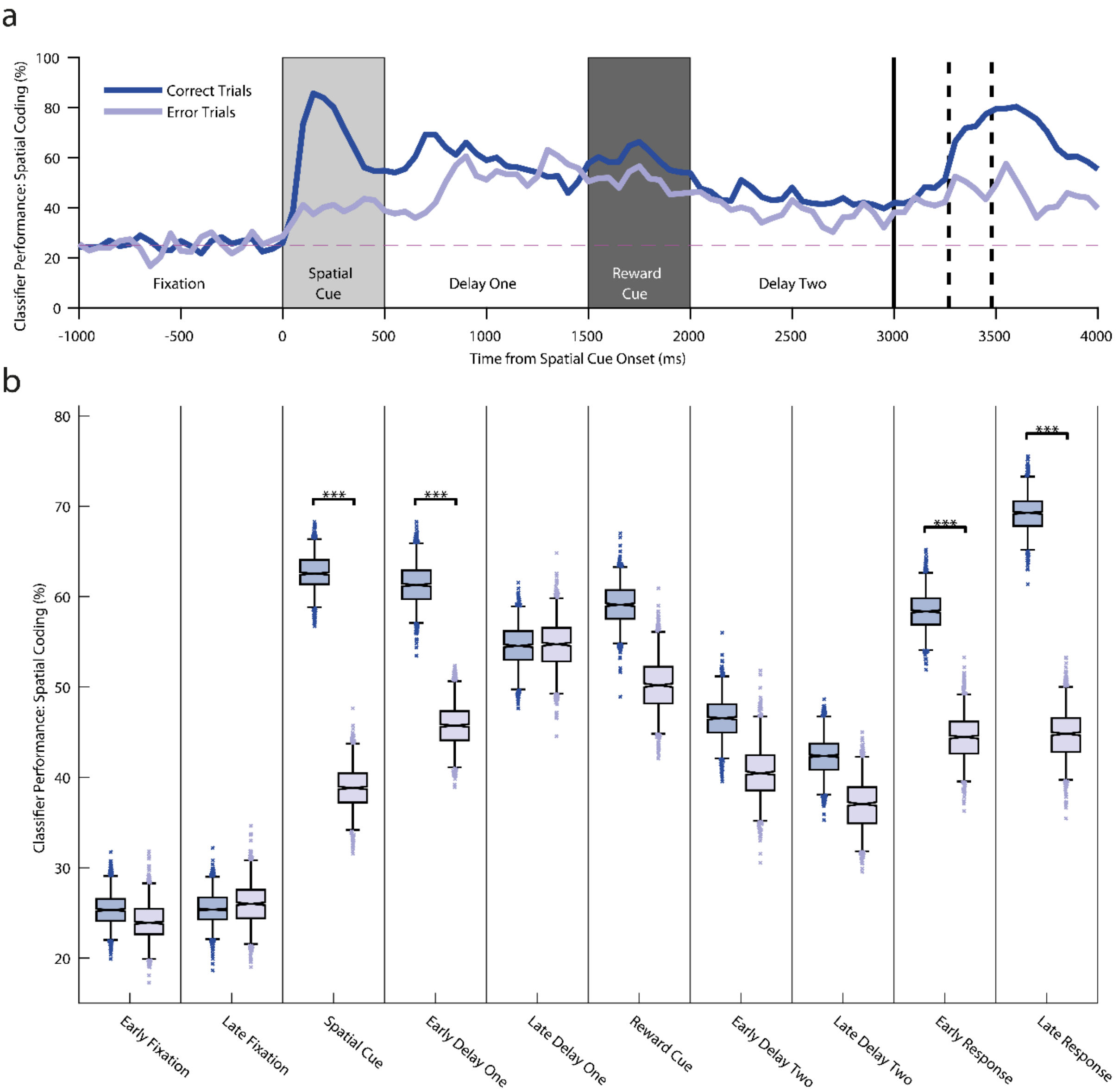

Supplementary Figure 1. Error trial analysis shows ventrolateral prefrontal cortex spatial activity on SR-trials is behaviourally relevant. a) The mean performance of a classifier (1000 permutations, see **Methods**) trained to decode spatial location through the trial. The decoder was trained on correct trials and tested on data from left-out correct trials (dark blue) or error trials (lighter blue). Dashed line shows chance-level performance. **b)** Comparison of classifier performance across the trial. Boxplots show the distribution of classifier accuracies, across permutations, within each epoch. Each epoch has a pair of boxplots; the left-side for correct trials (dark blue), and the right-side for error trials (lighter blue). The area contained within the whiskers of the boxplots represents the 95th percentile range of classifier performance. The box limits represent the upper and lower quartiles of the distribution. The central mark is the median of the distribution. Performance for correct and error trials was compared within each 500ms epoch using a Bonferroni-corrected bootstrap test (see **Methods**). On correct trials, the decoding accuracy is significantly higher during stimulus presentation, the initial delay, and around the time of response (***, p<0.001).

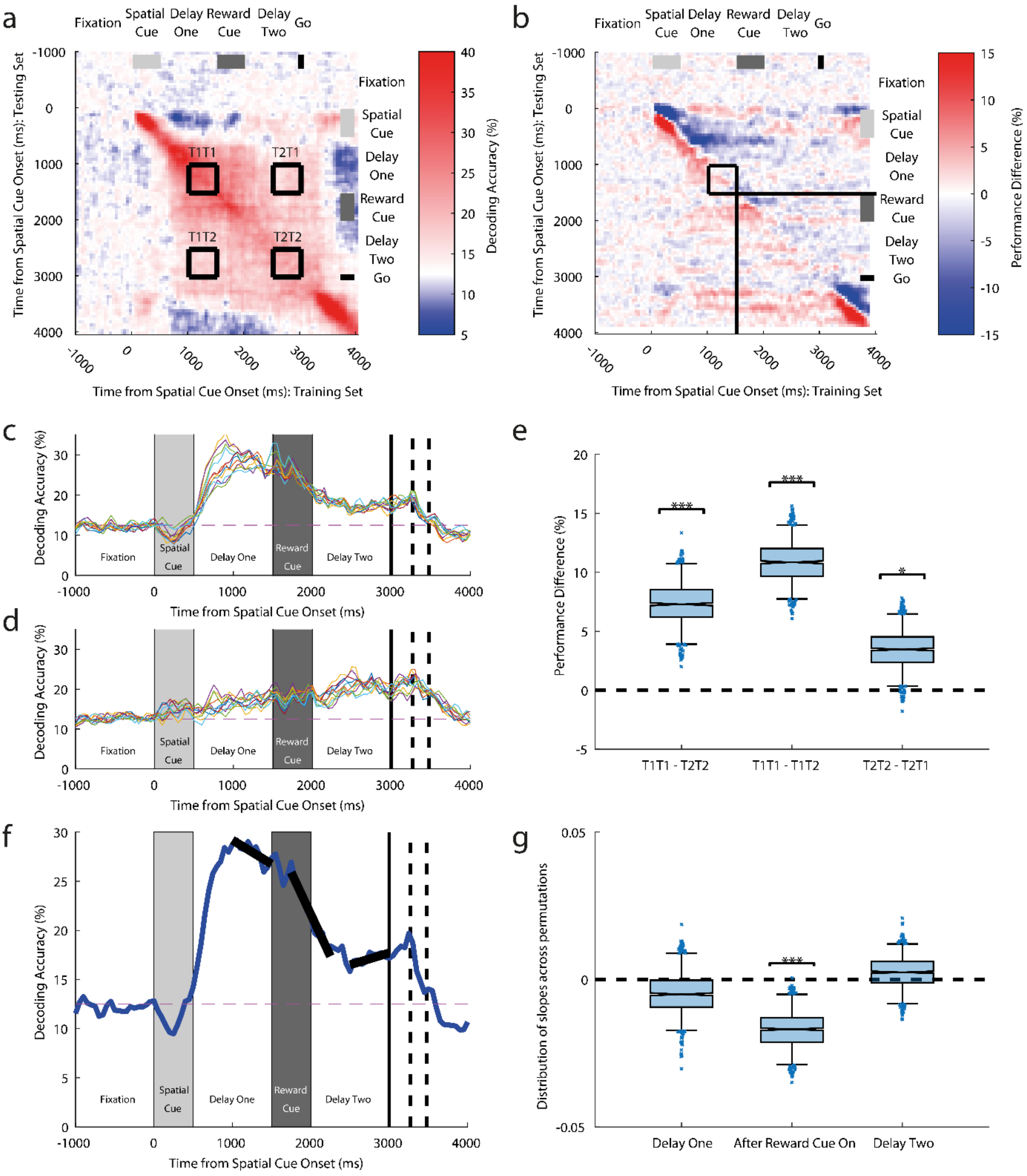

Supplementary Figure 2: Quantifying temporal evolution of ventrolateral prefrontal cortex spatial code on SR-trials. a) Cross-temporal decoding performance of spatial coding (see **Methods**). Annotated squares show time points used to compare decoding performance across epochs (T1T1 - Decoder trained in delay-one, tested in delay-one; T2T2 - Decoder trained in delay-two, tested in delay-two; T1T2 - Decoder trained in delay-one, tested in delay-two; T2T1 – Decoder trained in delay-two, tested in delay-one). **b)** Heatmap plotting the change in cross-temporal decoding performance between timepoint (t) and a bin three timepoints later. The annotated square shows T1T1, with the lines extending from it representing the onset of the reward cue. Reward cue onset appears to reduce decoding performance. **c)** Across-trial performance of all classifiers trained within the T1 window. **d)** Across-trial performance of all classifiers trained within the T2 window. **e)** Boxplot comparing the performance of different classifiers across 1000 permutations. T1T1-T2T2 (first bar) shows that spatial coding is significantly reduced from delay-one to delay-two. (***, p<0.001; *, p<0.05; bootstrap test, see **Methods**) **f)** Average performance of all classifiers trained within the T1 window. Black boxes show the gradient of the performance across time. Spatial coding is stable by the end of delay one, but a sharp drop follows the onset on the reward cue. **g)** Boxplots show the distributions of these gradients across permutations. The slope fitted following the reward cue onset is significantly negative (***, p=0.001; bootstrap test, see **Methods**), showing a decrease in spatial coding. The other slopes were not significantly different from zero. The area contained within the whiskers of the boxplots (**e, g**) represents the 95th percentile range of the distributions. The box limits represent the upper and lower quartiles of the distribution. The central mark is the median of the distribution.

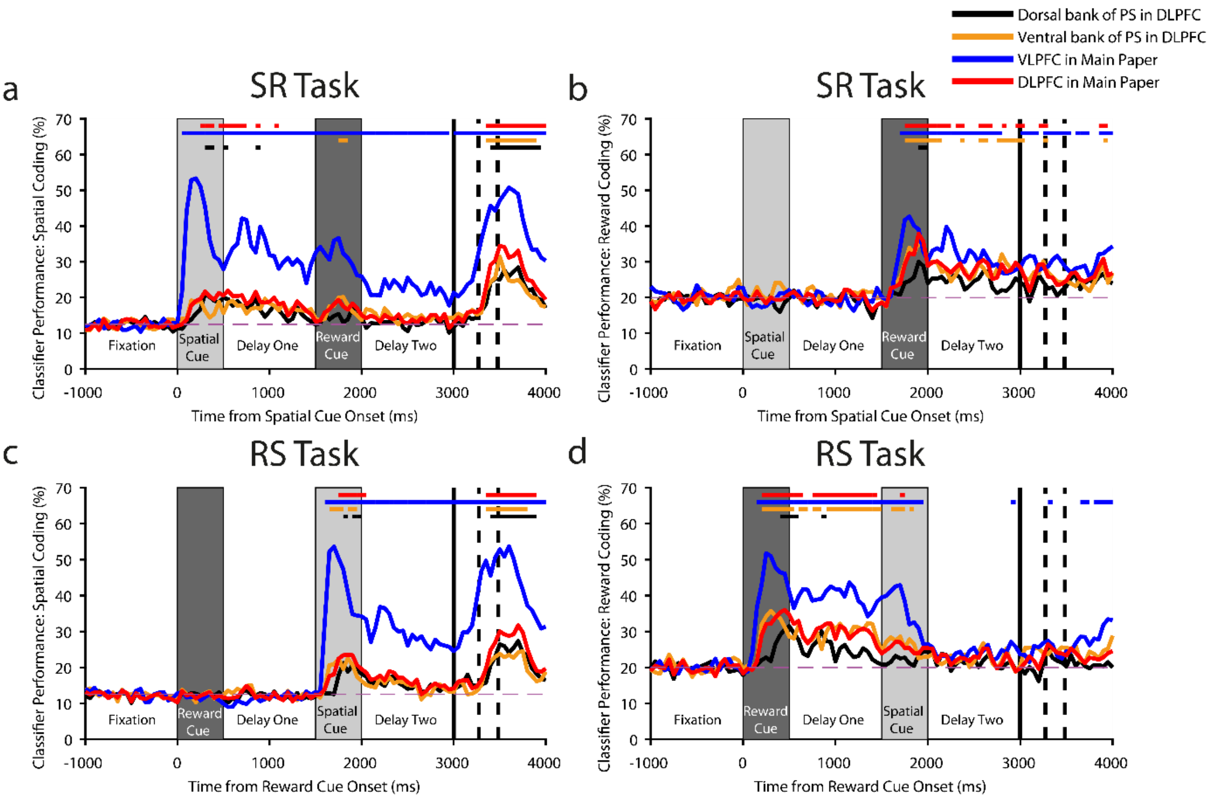

Supplementary Figure 3: Decoding performance of different populations of dorsolateral prefrontal cortex (DLPFC) and ventrolateral prefrontal cortex (VLPFC) neurons. In the main paper, we defined DLPFC as including all neurons within and around the dorsal bank of the Principal Sulcus (PS) in areas 9/46D and 46D (black line), and all neurons within and around the ventral bank of the PS in areas 46V and 9/46V (orange line). Note both 9/46D and 9/46V extend outside of the dorsal and ventral banks, respectively, by approximately 2mm. The Paxinos macaque monkey brain atlas[1] was used to define the brain area of each recorded neuron. The mean performance of a classifier (1000 permutations, see **Methods**) trained to decode each task feature (spatial location, **a** and **c**; reward level, **b** and **d**) are plotted for each neural population and trial type (SR-task, **a** and **b**; RS-task, **c** and **d**). The first solid vertical line signifies when subjects were cued to respond. The first and second dashed vertical lines represent the average timing of the subjects' saccade and the onset of reward respectively. Solid coloured horizontal lines represent significant encoding for the corresponding brain region (2.5th percentile of distribution>chance level, p<0.05, see **Methods**). The dashed magenta line represents chance level classifier performance.

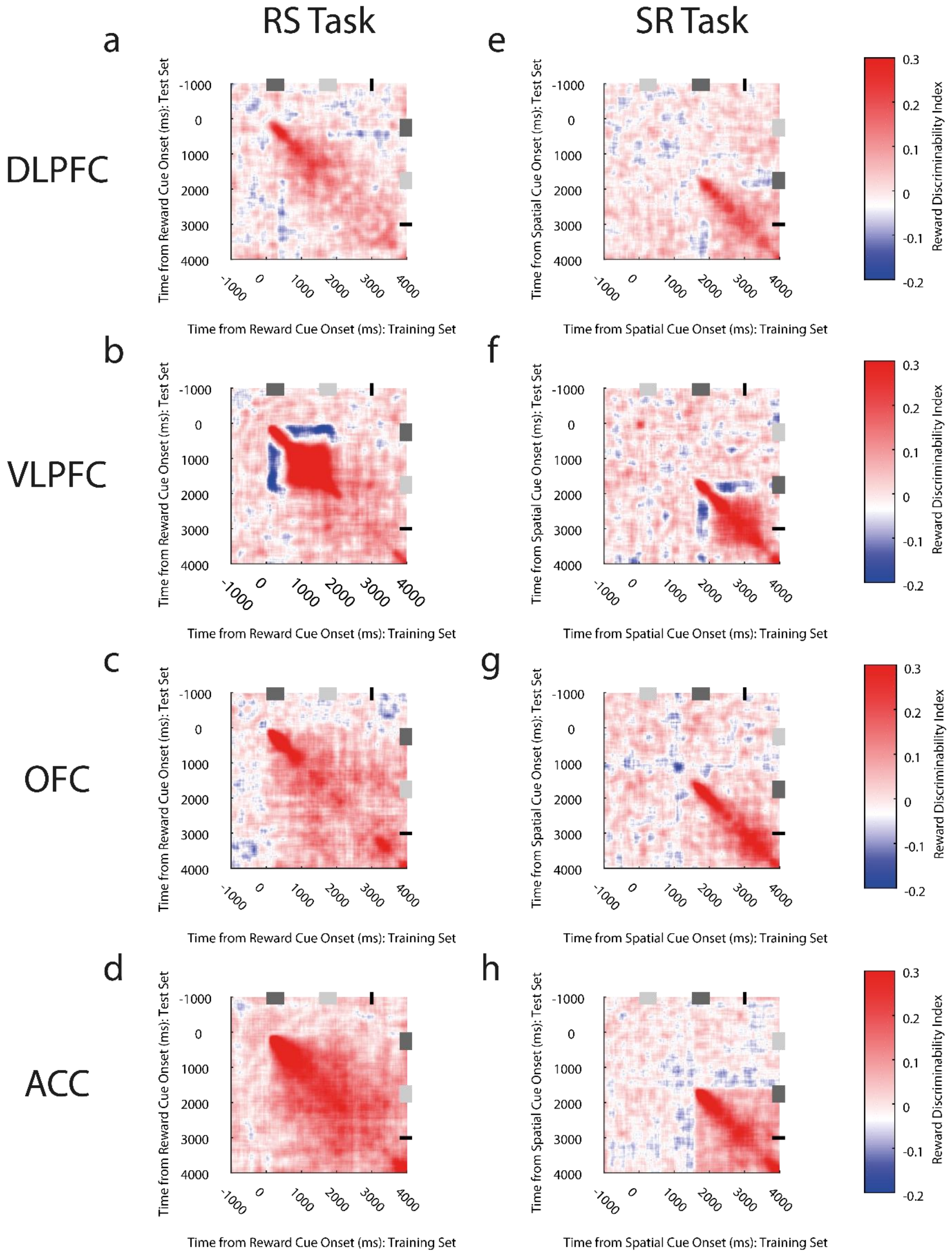

Supplementary Figure 4: Cross-temporal dynamics of reward selectivity by brain region and task. The cross-temporal decodability of reward size is plotted for DLPFC (**a**, **e**), VLPFC (**b**, **f**), OFC (**c**, **g**), and ACC (**d**, **h**) populations on RS (**a-d**) and SR (**e-h**) trials. All brain areas studied have neural activity representing reward size. Only VLPFC shows a reversal of reward tuning between the reward cue epoch and the subsequent delay. This feature of coding is present on both trial types. The dark grey and light grey boxes on the outer edges of the heatmaps represent the time when the reward cue and spatial cues respectively, were on the screen. The thin black line shows when the subject was cued to respond.

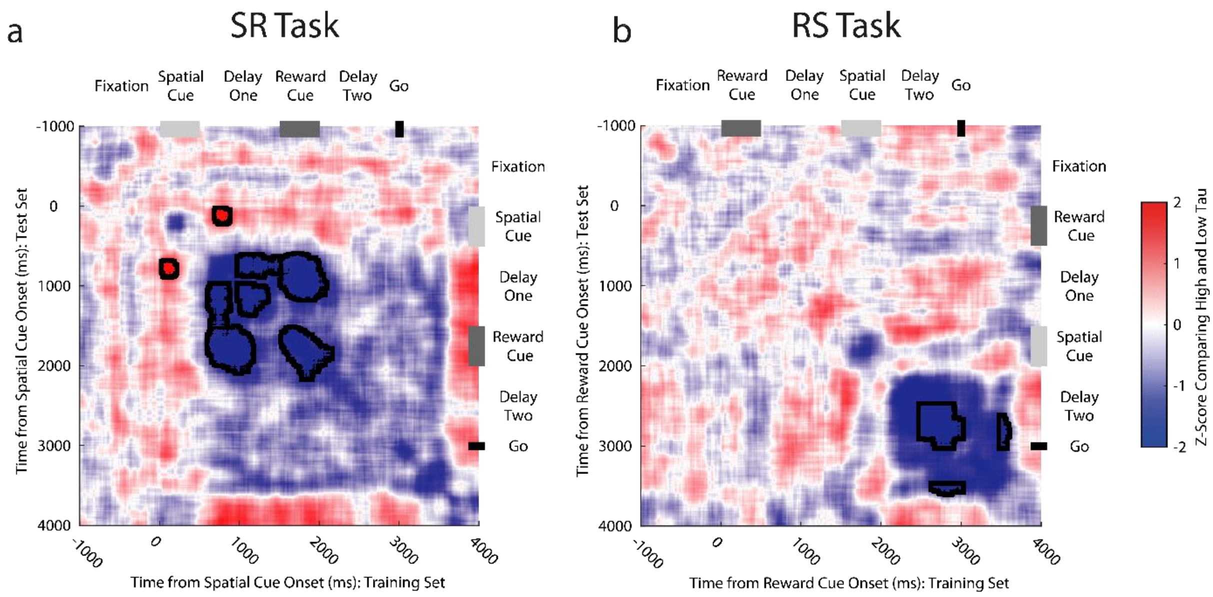

Supplementary Figure 5: Comparison of ventrolateral prefrontal cortex high and low-tau cross-temporal spatial coding. The strength of cross-temporal spatial coding is compared between the two populations for **a**) SR-trials and **b**) RS-trials. Negative z-scores illustrate stronger coding in the high-tau population. **a)** Coding of spatial location is more stable for the high-tau population during the first delay (largest cluster, p = 0.0050; cluster-based permutation test; see **Methods**). There was also stronger coding between delay-one and the reward cue of SR-trials (largest cluster, p < 0.0001; cluster-based permutation test), and during the reward cue of SR-trials (largest cluster, p = 0.0072; cluster-based permutation test). There was also a stronger switch in coding between the spatial cue and the first delay in high-tau cells (largest cluster, p = 0.0152; cluster-based permutation test). **b)** On RS-trials, there is more stable coding in high-tau cells during delay-two (largest cluster, p = 0.0388; cluster-based permutation test), as well as between this time and the reward onset (largest cluster, p = 0.0241; cluster-based permutation test). Black lines encircling areas of strong dissimilarities in coding indicate a significant difference in cross-temporal stability between high-tau and low-tau populations (p<0.05; cluster-based permutation test).

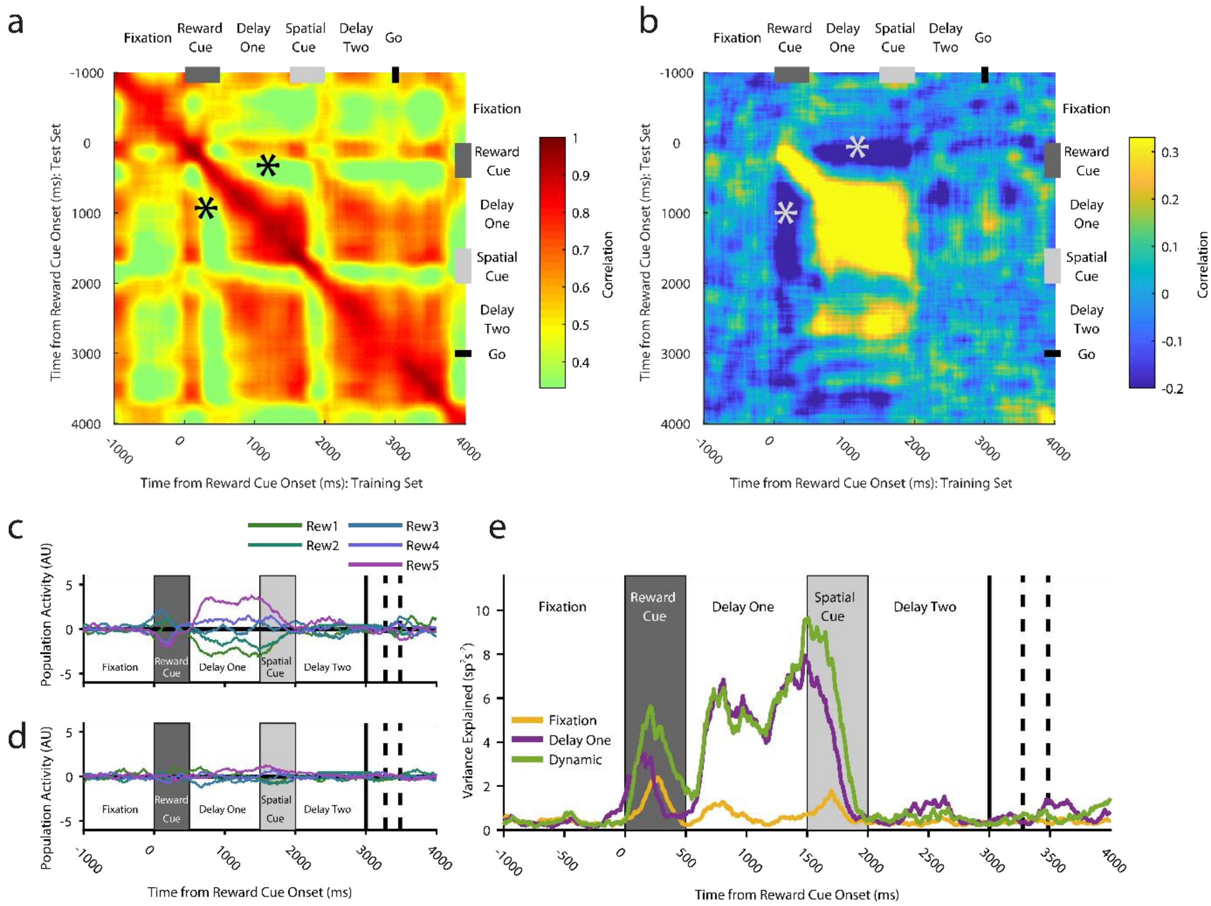

Supplementary Figure 6: VLPFC high time-constant population reverses its reward coding between cue presentation and the subsequent delay. a) Within-condition correlation of neural firing across time for RS-trials. All bins are positively correlated with each other, suggesting neural firing is stable across time. Note positive correlation between cue period and delay (asterisk). **b)** Within-condition correlation analysis where activity for each neuron was demeaned across each of the reward sizes (see **Methods**). There now exists a negative correlation between the time of the reward cue presentation and the first delay (asterisk). **c-d)** Reversal of VLPFC high time-constant reward tuning between cue and delay. A mnemonic subspace was defined with time-averaged delay-one activity. The across-trial firing for each condition was projected back onto the first (**c**) and second (**d**) principal axes of this subspace. While the conditions remain well separated on the first principal axis during delay-one, the subspace does not generalise well into delay-two as activity from the different conditions converges. At the time of the cue, the conditions appear separable, but in the reverse configuration from that during delay-one. **e)** The stimulus variance captured by three different subspaces is displayed. The fixation subspace is defined with time-averaged activity in the 1000ms before cue presentation. This should represent a chance-level amount of variance explained. The delay-one subspace is defined with time-averaged activity from 500ms to 1500ms after cue presentation. The dynamic subspace is defined separately at each individual time point. The dynamic subspace explains a much greater amount of variance during the cue period, illustrating that there is little consistency in the activity patterns between cue and delay epochs. However, the delay-one subspace captures as much variance as the dynamic subspace during delay-one, suggesting the VLPFC high-tau population activity has settled to a stable code by this point.

1 Paxinos, G., Huang, X. F. & Toga, A. W. *The Rhesus Monkey Brain in Stereotaxic Coordinates*. (Academic Press, 2000).
